# On Bayesian approach to composite Pareto models

**DOI:** 10.1371/journal.pone.0257762

**Published:** 2021-09-23

**Authors:** Muhammad Hilmi Abdul Majid, Kamarulzaman Ibrahim

**Affiliations:** Department of Mathematical Sciences, Faculty of Science and Technology, Universiti Kebangsaan Malaysia, Bangi, Selangor, Malaysia; Universita degli Studi di Catania, ITALY

## Abstract

In data modelling using the composite Pareto distribution, any observations above a particular threshold value are assumed to follow Pareto type distribution, whereas the rest of the observations are assumed to follow a different distribution. This paper proposes on the use of Bayesian approach to the composite Pareto models involving specification of the prior distribution on the proportion of data coming from the Pareto distribution, instead of assuming the prior distribution on the threshold, as often done in the literature. Based on a simulation study, it is found that the parameter estimates determined when using uniform prior on the proportion is less biased as compared to the point estimates determined when using uniform prior on the threshold. Applications on income data and finance are included for illustrative examples.

## 1 Introduction

Size-type data may exhibit heavy upper tail property that must be taken into account in statistical modelling. Pareto type distributions have been used successfully to model the distribution of the upper tail [1, 2] but these distributions can only model the observations above a certain threshold and do not take into account of any observations below the threshold value. In this case, a separate distribution is required for describing any observations below the threshold value.

Composite Pareto models are a family of distribution models that splice the observations into two parts: observations below a threshold value and observations above the threshold, where the threshold is treated as a parameter. In these models, any observations with values lower than or equal to the threshold are distributed according to a lower data distribution model whereas any observations with values higher than the threshold are assumed to follow a Pareto type distribution. The probability density function (pdf) for the composite Pareto models can be written as:

$$\begin{array}{c} f\left(x|\theta \right)=\{\begin{array}{cc} c_{1}f_{1}\left(x|\theta \right),\; & x\leq \tau ,\\ c_{2}f_{2}\left(x|\theta \right),\; & x>\tau , \end{array} \end{array}$$

where *c*_1_ and *c*_2_ are the mixing weights of the components in the composite model, *τ* is the threshold value, *f*_1_(*x*|***θ***) is the distribution model for observations below the threshold and *f*_2_(*x*|***θ***) is the Pareto model for observations above the threshold. This property of having separate models for observations below and above the threshold value is desirable when the upper tail observations are heavily skewed or believed to be distributed differently compared to the rest of the observations in the data. It has found many applications including in the area of insurance [3], survival data [4], internet traffic data [5] and ecology [6].

In this paper, a general implementation of Bayesian approach to the composite Pareto models with respect to specifying prior information on the proportion of observations coming from the Pareto tail is outlined and discussed. Although other literatures have discussed and used Bayesian analysis for composite Pareto models [7, 8], their approach are tied to specifying prior information on the threshold parameter, not on the proportion of data in the Pareto tail as proposed in this paper. Additionally, through simulation study, we demonstrate that using uniform prior on the proportion leads to less biased point estimates, when compared to using uniform prior on the threshold parameter.

This paper is outlined as follows. Section 2 gives a brief summary of the composite Pareto models. Section 3 provides a discussion on determining threshold value when the other parameters are given. Then prior distributions for composite Pareto models are discussed in Section 4. In Section 5, a simulation study is presented to compare the performance of point estimates based on uniform prior on the threshold versus uniform prior on the proportion of data coming from Pareto tail. We apply the composite Pareto models to the Malaysian household income data and NASDAQ-100 stock index in Section 6. Section 7 concludes the paper.

## 2 Composite Pareto models

Composite Pareto models divide the data into two parts: data with values lower than a threshold value, hereafter is termed as the lower data, and data with values higher than the threshold, called the upper data. The models assume that the distributions for the lower and upper data are different with the upper data following a Pareto distribution. Typically, a lighter tail distribution is used for the lower data, including truncated lognormal, truncated gamma, or truncated Weibull distributions.

The composite Pareto models were first introduced by Cooray and Ananda [3]. In their work, the lower data is distributed using lognormal distribution while the upper data is distributed using Pareto type I distribution. However, as noted by Scollnik [9], the model introduced is limited since the mixing proportion of data coming from Pareto distribution is fixed. Scollnik [9] has freed the mixing proportion and improves the composite model by considering Pareto type II distribution for the upper tail data. Since then, there have been many other composite Pareto models proposed, including Weibull-Pareto composite model [10–12], gamma-Pareto composite model [12], exponential-Pareto composite model [13], inverse gamma-Pareto composite model [8] and composite Stoppa model [14].

### 2.1 Composite Pareto I models

Let ***θ*** be the collection of all parameters and let *h*(*x*|***η***) be a probability density function (pdf) with a collection of parameters ***η*** and cumulative distribution function (cdf) *H*(*x*|***η***). We call this the lower data distribution. The composite Pareto I model has the following pdf:

$$\begin{array}{c} f\left(x|\theta \right)=\{\begin{array}{cc} \left(1-\rho \right)\frac{h(x|\eta )}{H(\tau |\eta )},\; & x\leq \tau ,\\ \rho f_{P1}\left(x|\tau ,\alpha \right),\; & x>\tau , \end{array} \end{array}$$ (1)

where *ρ* > 0 is the proportion of data coming from Pareto I distribution with pdf *f*_*P*1_(*x*|*τ*, *α*), *τ* > 0 is the threshold that separates the lower and upper data, and *α* > 0 is the tail index for the Pareto type I distribution. Note that

$$\begin{array}{c} f_{P1}\left(x|\tau ,\alpha \right)=\frac{\alpha \tau^{\alpha }}{x^{\alpha +1}},\;x>\tau . \end{array}$$

In the pdf (1), any observation smaller than the threshold *τ* is distributed according to the lower data distribution, and any observation bigger than *τ* is distributed according to the Pareto type I distribution. Note that the pdf (1) may not be continuous and differentiable at *τ*. To make it continuous, we can specify

$$\begin{array}{c} \rho =\frac{\tau h(\tau |\eta )}{\tau h(\tau |\eta )+\alpha H(\tau |\eta )}. \end{array}$$

And to further make it differentiable, we let

$$\begin{array}{c} \alpha =-1-\frac{\tau h^{\prime }\left(\tau |\eta \right)}{h(\tau |\eta )}, \end{array}$$ (2)

condition on *τh*′(*τ*|***η***) < −*h*(*τ*|***η***) so that *α* > 0. This simplifies the condition on *ρ* to

$$\begin{array}{c} \rho =\psi_{1}\left(\tau ,\eta \right)=\frac{\tau h{\left(\tau |\eta \right)}^{2}}{\tau h{\left(\tau |\eta \right)}^{2}-H\left(\tau |\eta \right)\left[h\left(\tau |\eta \right)+\tau h^{\prime }\left(\tau |\eta \right)\right]}. \end{array}$$ (3)

### 2.2 Composite Pareto II models

Composite Pareto II models are similar to composite Pareto I models except for the use of Pareto type II distribution for the upper data. As before, let ***θ*** be the collection of all parameters, *h*(*x*|***η***) be a pdf with its cdf *H*(*x*|***η***), and ***η*** be a collection of parameters associated with *h*(*x*|***η***). Then the composite Pareto II models have the following pdf:

$$\begin{array}{c} f\left(x|\eta \right)=\{\begin{array}{cc} \left(1-\rho \right)\frac{h(x|\eta )}{H(\tau |\eta )},\; & x\leq \tau ,\\ \rho f_{P2}\left(x|\tau ,\alpha ,\beta \right),\; & x>\tau , \end{array} \end{array}$$ (4)

where *ρ* > 0 is the proportion of data coming from Pareto type II distribution with pdf *f*_*P*2_(*x*|*τ*, *α*, *β*), *τ* > 0 is the threshold that separates lower and upper data distributions, whereas *α* > 0 and *β* > 0 are the tail index and scale parameters of the Pareto type II distribution, respectively. The pdf of the Pareto type II is:

$$\begin{array}{c} f_{P2}\left(x|\tau ,\alpha ,\beta \right)=\frac{\alpha }{\beta }(1+\frac{x-\tau }{\beta }{)}^{-\alpha -1},\;x>\tau . \end{array}$$

The Pareto type II distribution is also a special case of generalized Pareto distribution with positive scale parameter.

In this case, observations with value less than *τ* is assumed to follow the lower data distribution while any observation greater than *τ* follows the Pareto type II distribution. To make pdf (4) continuous at *τ*, we can specify

$$\begin{array}{c} \rho =\frac{\beta h(\tau |\eta )}{\beta h(\tau |\eta )+\alpha H(\tau |\eta )}. \end{array}$$

And for differentiability condition, let

$$\begin{array}{c} \beta =-\frac{\left(\alpha +1)h(\tau |\eta \right)}{h^{\prime }\left(\tau |\eta \right)}, \end{array}$$ (5)

condition to *h*′(*τ*|***η***) < 0. This simplifies the condition on *ρ* to

$$\begin{array}{c} \rho =\psi_{2}\left(\tau ,\eta ,\alpha \right)=\frac{\left(\alpha +1\right)h{\left(\tau |\eta \right)}^{2}}{\left(\alpha +1\right)h{\left(\tau |\eta \right)}^{2}-\alpha h^{\prime }\left(\tau |\eta \right)H\left(\tau |\eta \right)}. \end{array}$$ (6)

The twice differentiability property can be satisfied by specifying the value of *α*. However it is not a focus here due to it being too restrictive [9]. In this paper, focus will be given to the composite models with continuous and differentiable pdf. Going forward, when composite Pareto models are mentioned, we refer to composite Pareto models with smooth pdf, unless specified otherwise.

### 2.3 Bulk models

The previous subsections deal with the general case of composite Pareto models. In the general model, the parameter *ρ* is free, or is specified to satisfy the continuity and differentiability conditions. However, in some cases, we may specify:

$$\begin{array}{c} \rho =1-H(\tau |\eta ) \end{array}$$ (7)

for simplification. This specification is referred to as the “bulk model based tail fraction” by Scarrot [15] and is commonly found in the extreme value literature. The term “bulk model” refers to the lower data distribution that is assumed to covers the bulk of the observations when compared to the Pareto distribution. It was first proposed by Behrens et al. [16] as a way to estimate the threshold value for extreme value data and improvements have been made since then [17–19]. In this paper, we refer composite Pareto models with specification (7) as simply the bulk models.

Historically, the bulk models are developed separately with the general composite Pareto models in the previous subsections. There are two main differences in the development of this two models. First, the general composite Pareto models appear in the actuarial science literature while the bulk models appear in the extreme value literature. Second, the general composite Pareto models typically use frequentist approach to estimate the parameters while the bulk models use Bayesian approach for parameter estimation. Readers interested in the bulk models can refer to [15] which gives an outline on the developments and bibliography related to the bulk models. We include this special case of composite Pareto models since the results shown in this paper still apply to the bulk models.

For bulk models with Pareto I tail, the pdf can be written as:

$$\begin{array}{c} f\left(x|\theta \right)=\{\begin{array}{cc} h(x|\eta ),\; & x\leq \tau ,\\ {}[1-H(\tau |\eta )]f_{P1}\left(x|\tau ,\alpha \right),\; & x>\tau . \end{array} \end{array}$$ (8)

Whereas for bulk models with Pareto II tail, the pdf is:

$$\begin{array}{c} f\left(x|\theta \right)=\{\begin{array}{cc} h(x|\eta ),\; & x\leq \tau ,\\ {}[1-H(\tau |\eta )]f_{P2}\left(x|\tau ,\alpha ,\beta \right),\; & x>\tau . \end{array} \end{array}$$ (9)

Note that in the literature covering the bulk models, the continuity and differentiability of the pdf are usually not imposed. This is to allow for more flexibility to model the lower data distribution. In some cases, researchers may even use semi-parametric or nonparametric approach for specifying *h*(*x*|***η***) [17, 20, 21].

However, if desired, the continuity condition for bulk models can be imposed by specifying

$$\begin{array}{c} \alpha =\frac{\tau h(\tau |\eta )}{1-H(\tau |\eta )}, \end{array}$$ (10)

or

$$\begin{array}{c} \beta =\frac{\alpha [1-H(\tau |\eta )]}{h(\tau |\eta )}, \end{array}$$ (11)

for bulk models with Pareto I and Pareto II tail, respectively.

Differentiability condition is more complicated here as it requires specifying parameter in the lower data distribution. For example, the pdf (8) is differentiable when Eq (10) is satisfied and

$$\begin{array}{c} h^{\prime }\left(\tau |\eta \right)=-\frac{h(\tau |\eta )}{\tau }[1+\frac{\tau h(\tau |\eta )}{1-H(\tau |\eta )}]. \end{array}$$

If *h*(*x*|***η***) is a lognormal distribution with log-mean *μ* and log-variance *σ*^2^, then the above condition simplifies to

$$\begin{array}{c} \frac{\text{log}\;\tau -\mu }{\sigma }=\frac{\text{exp}\{-\frac{1}{2}(\frac{\text{log}\;\tau -\mu }{\sigma }{)}^{2}\}}{\sqrt{2\pi }(1-\Phi (\frac{\text{log}\;\tau -\mu }{\sigma }))}, \end{array}$$

which does not have a solution for finite *σ*. Therefore in this case the differentiability condition cannot be met.

Similarly, the pdf (9) is differentiable when Eq (11) is satisfied and

$$\begin{array}{c} \alpha =-\frac{h{\left(\tau |\eta \right)}^{2}}{h{\left(\tau |\eta \right)}^{2}+h^{\prime }\left(\tau |\eta \right)\left[1-H\left(\tau |\eta \right)\right]} \end{array}$$

condition to *h*(*τ*|***η***)^2^ + *h*′(*τ*|***η***)[1 − *H*(*τ*|***η***)] < 0. However, this condition may not be met by all lower data distributions. For example, if the lower data distribution is lognormal, then it can be proven that the condition cannot be met unless the log-variance is greater than 1.

## 3 Threshold as a function of other parameters

The main contribution of this paper is to propose using prior information on the proportion of observations coming from Pareto distribution, *ρ*, rather than on the threshold value, *τ*, which is often suggested by other literatures. To achieve this objective, it is important to first study the relationship between *ρ* and *τ*. For the composite Pareto models, under continuity condition, *ρ* is a function of other parameters including *τ*. For example, for smooth composite Pareto I models, *ρ* = *ψ*_1_(*τ*, ***η***) as shown in Eq (3) and for smooth composite Pareto II models, *ρ* = *ψ*_2_(*τ*, ***η***, *α*) as shown in Eq (6).

For composite Pareto I model, if prior information on *τ* is used in Bayesian estimation, then *ρ* is a function of random variables *τ* and ***η*** as in Eq (3). However, if we want to use prior information on *ρ* instead, then we would have to invert the function so that *τ* can be determined for given values of *ρ* and ***η***. Therefore we can write

$$\begin{array}{c} \tau \left(\rho ,\eta \right)=\{\tau :\rho =\psi_{1}\left(\tau ,\eta \right)\;\text{and}\;\tau h^{\prime }\left(\tau |\eta \right)<-h\left(\tau |\eta \right)\} \end{array}$$ (12)

Similarly for composite Pareto II model, *ρ* is a function of *τ*, ***η*** and *α* as in Eq (6). The function must be inverted for us to compute the value of *τ* when *ρ*, ***η*** and *α* are given. We write

$$\begin{array}{c} \tau \left(\rho ,\eta ,\alpha \right)=\{\tau :\rho =\psi_{2}\left(\tau ,\eta ,\alpha \right)\;\text{and}\;h^{\prime }\left(\tau |\eta \right)<0\} \end{array}$$ (13)

Note that the solution to these two functions above may require numerical computation for estimation.

However, the solution for Eqs (12) and (13) may not exist for some choices of lower data distributions and parameters. For example, consider a normal-Pareto II composite model where *h*(*x*|***η***) is the pdf of normal distribution with mean −1 and variance 1, and suppose *α* is set to 2. Then the highest possible value of *ρ* for this model is approximately 0.3014 when *τ* → 0. Therefore, in this case, there will be no solution to Eq (13) when finding the value of *τ* for *ρ* > 0.3014.

There are two ways that can be used to show that the solution always exists for some lower data distributions. First, if the pdf is only defined for *x* > 0, it is enough to show that *xh*′(*x*|***η***) < −*h*(*x*|***η***) for all *x* > 0 for Eq (12), or that *h*′(*x*|***η***) < 0 for all *x* > 0 for Eq (13). In this case, *ρ* = 1 when *τ* → 0, *ρ* = 0 when *τ* → ∞, and *ρ* is a continuous function of *τ* and other parameters. By the intermediate value theorem, the solution must exists. The second method to show that the solution exists is to show that the there exists x* > 0 such that x*h′(x*|***η***) = −*h*(x*|***η***) for composite Pareto I model, or *h*′(x*|***η***) = 0 for composite Pareto II model. In this case, there will be a smooth function of *ρ* given *τ* and other parameters going from *ρ* = 1 (when *τ* → x*) to *ρ* = 0 (when *τ* → ∞). Therefore again by the intermediate value theorem, the solution to Eqs (12) or (13) must exists.

Unfortunately, the uniqueness of solution to the above equations is not guaranteed for all lower data distributions. There may be multiple values of *τ* that satisfy the equations even when using the same value of *ρ*, ***η*** and *α*, especially when *h*(*x*|***η***) is multimodal. For example, suppose the lower data distribution is a two-component lognormal mixture with $h\left(x|\eta \right)=r_{1}f_{LN}\left(x|\mu_{1},\sigma_{1}^{2}\right)+r_{2}f_{LN}\left(x|\mu_{2},\sigma_{2}^{2}\right)$ and the parameters ***r*** = (0.5, 0.5), ***μ*** = (5, 7), ***σ*** = (0.6, 0.3) where *f*_*LN*_(*x*|*μ*, *σ*^2^) is the pdf of lognormal distribution with log-mean *μ* and log-variance *σ*^2^. Then in this case there will be three solutions to Eq (12) using *ρ* = 0.2: *τ* = 280.69, 476.23, or 1355.44 with *α* = 1.768, 0.526, and 2.357, respectively.

The value of *τ* is important to be determined as it is required in the calculation of the likelihood function. When unique solution of *τ* based on Eqs (12) or (13) is guaranteed, the solution of *τ* can be used for calculating the likelihood function and to estimate the posterior distribution in Bayesian analysis. On the other hand, when there are multiple values of *τ* that satisfy Eqs (12) or (13), we cannot simply use any of the solutions without valid reasoning. Therefore, there is a need for a method to select which *τ* is most suitable, and this will be discussed in the following subsections.

For the bulk model mentioned in Section 2.3, it is assumed that *ρ* = 1 − *H*(*τ*|***η***) or inversely, *τ* = *H*^−1^((1 − *ρ*)|***η***). Then in this case, there is a unique solution of *τ* given the values of *ρ* and ***η*** since *H*^−1^((1 − *ρ*)|***η***) is a strictly monotonic function. We can then use the prior on *ρ* rather than *τ* for Bayesian estimation without much problem due to the uniqueness of *τ* when the values of other parameters are given.

### 3.1 Unique solution of the threshold

Fortunately, the uniqueness of the solutions to Eqs (12) and (13) can be proven for many commonly used lower data distributions including gamma, lognormal, normal, and Weibull distributions. These distributions have the property that *h*′(*x*|***η***) = *h*(*x*|***η***)*g*(*x*|***η***) for some function *g*(*x*|***η***). The following two theorems provide a way to determine for the uniqueness of the solution to (12) and (13) with proofs shown in S1 Appendix.

**Theorem 1**. *Suppose*

$$h^{\prime }\left(x|\eta \right)=h\left(x|\eta \right)g\left(x|\eta \right)$$

*for some function g*(*x*|***η***). *Suppose the solution to*

$$\tau \left(\eta ,\rho \right)=\{\tau :\rho =\frac{\tau h{\left(\tau |\eta \right)}^{2}}{\tau h{\left(\tau |\eta \right)}^{2}-H\left(\tau |\eta \right)\left[h\left(\tau |\eta \right)+\tau h^{\prime }\left(\tau |\eta \right)\right]}\;\text{and}\;\tau h^{\prime }\left(\tau |\eta \right)<-h\left(\tau |\eta \right)\}$$

*exists*. *If g*(*τ*|***η***) + *τg*′(*τ*|***η***) < 0 *for all τ that satisfies h*(*τ*|***η***) + *τh*′(*τ*|***η***) < 0, *then the solution above must be unique, and the relationship between τ and ρ is one-to-one*.

**Theorem 2**. *Suppose*

$$h^{\prime }\left(x|\eta \right)=h\left(x|\eta \right)g\left(x|\eta \right)$$

*for some function g*(*x*|***η***). *Suppose that the solution to*

$$\tau \left(\rho ,\eta ,\alpha \right)=\{\tau :\rho =\frac{\left(\alpha +1\right)h{\left(\tau |\eta \right)}^{2}}{\left(\alpha +1\right)h{\left(\tau |\eta \right)}^{2}-\alpha h^{\prime }\left(\tau |\eta \right)H\left(\tau |\eta \right)}\;\text{and}\;h^{\prime }\left(\tau |\eta \right)<0\}$$

*exists*. *If h*′(*τ*|***η***) + *H*(*τ*|***η***)[*g*′(*τ*|***η***) − *g*(*τ*|***η***)^2^] < 0 *for all τ that satisfies h*′(*τ*|***η***) < 0, *then the solution above must be unique, and the relationship between τ and ρ is one-to-one*.

Theorem 1 can be used for the composite Pareto I models while Theorem 2 is useful for composite Pareto II models when checking for the uniqueness of the solution to Eqs (12) and (13). Under Theorem 1, for lower data distribution with *h*′(*x*|***η***) = *h*(*x*|***η***)*g*(*x*|***η***) for some function *g*(*x*|***η***), it is enough to show that *g*(*τ*|***η***) + *τg*′(*τ*|***η***) < 0 to determine the uniqueness of solution to Eq (12). For composite Pareto II models, it is enough to show that *h*′(*τ*|***η***) + *H*(*τ*|***η***)[*g*′(*τ*|***η***) − *g*(*τ*|***η***)^2^] < 0 to show the uniqueness of the solution to Eq (13). Following Theorem 2, the condition to determine the uniqueness of the solution can be further simplified as shown in the following corollary.

**Corollary 1**. *Suppose h*′(*x*|***η***) = *h*(*x*|***η***)*g*(*x*|***η***) *for some function g*(*x*|***η***). *If g*′(*τ*|***η***) − *g*(*τ*|***η***)^2^ < 0, *then the solution to*

$$\tau \left(\rho ,\eta ,\alpha \right)=\{\tau :\rho =\frac{\left(\alpha +1\right)h{\left(\tau |\eta \right)}^{2}}{\left(\alpha +1\right)h{\left(\tau |\eta \right)}^{2}-\alpha h^{\prime }\left(\tau |\eta \right)H\left(\tau |\eta \right)}\;\text{and}\;h^{\prime }\left(\tau |\eta \right)<0\}$$

*must be unique*

As an example, suppose *h*(*x*|***η***) = *f*_*LN*_(*x*|*μ*, *σ*^2^) be the pdf of a lognormal distribution with log-mean *μ* and log-variance *σ*^2^. Then

$$h^{\prime }\left(x|\eta \right)=h\left(x|\eta \right)[-\frac{1}{x}(1+\frac{\text{log}\;x-\mu }{\sigma^{2}})].$$

Therefore we can write

$$g(x|\eta )=-\frac{1}{x}(1+\frac{\text{log}\;x-\mu }{\sigma^{2}})$$

$$g^{\prime }\left(x|\eta \right)=\frac{1}{x^{2}}(1+\frac{\text{log}\;x-\mu }{\sigma^{2}})-\frac{1}{x^{2}\sigma^{2}}$$

It follows that $g\left(x|\eta \right)+xg^{\prime }\left(x|\eta \right)=-\frac{1}{x\sigma^{2}}<0$. Therefore using Theorem 1, the solution to Eq (12) is unique and there is no need to check for multiple solutions to *τ* for given values of *ρ* and other parameters when using lognormal-Pareto I composite model.

Additionally, let *z* = log(*x*) − *μ*. Let *φ*(*x*) and Φ(*x*) be the pdf and cdf of the standard normal distribution, respectively. It can be shown that

$$\begin{array}{cc} h^{\prime }\left(x|\eta \right)+H\left(x|\eta \right)[g^{\prime }\left(x|\eta \right) & +g{\left(x|\eta \right)}^{2}]\\ & =-\frac{1}{x^{2}\sigma }\varphi (\frac{z}{\sigma })[1+\frac{z}{\sigma^{2}}]-\frac{1}{x^{2}}\Phi (\frac{z}{\sigma })[\frac{z^{2}}{\sigma^{4}}+\frac{z}{\sigma^{2}}+\frac{1}{\sigma^{2}}]\\ & =-\frac{\Phi (z/\sigma )}{x^{2}\sigma^{4}}[\frac{\varphi (z/\sigma )}{\Phi (z/\sigma )}(\sigma^{3}+z\sigma )+z^{2}+z\sigma^{2}+\sigma^{2}]. \end{array}$$ (14)

If *z* ≥ 0, then the equation above is negative and the condition for Theorem 2 is satisfied. However if *z* < 0, we would have to use the property of Mill’s ratio to show that the condition is still valid. Gordon [22] has proven that the Mill’s ratio

$$\frac{1-\Phi (x)}{\varphi (x)}\leq \frac{1}{x}$$

for *x* > 0. If *z* < 0, it follows that

$$\frac{\varphi (z/\sigma )}{\Phi (z/\sigma )}=\frac{\varphi (-z/\sigma )}{1-\Phi (-z/\sigma )}\geq -\frac{z}{\sigma }$$

and

$$\frac{\varphi (z/\sigma )}{\Phi (z/\sigma )}(\sigma^{3}+z\sigma )+z^{2}+z\sigma^{2}+\sigma^{2}\geq \sigma^{2}>0$$

This combines with Eq (14) satisfies the condition for Theorem 2. Therefore, there will not be instances of multiple *τ* solution for (13) and prior information on *ρ* can be used without problem for lognormal-Pareto II composite model.

### 3.2 Multiple solutions of the threshold

As mentioned previously, it is possible to have multiple solutions to *τ* given the values of *ρ* and other parameters. When such incidence happens, an analyst have the choice to either avoid the problem, or choose one of the solutions to *τ*. The former choice can be done by either using prior information on *τ*, use lower data distribution with proven solution uniqueness as shown in the previous subsection, or to use the bulk models.

If the analyst opt to select one of the solutions to Eqs (12) or (13), a naive way to select the value of *τ* out of the multiple solutions is to choose one at random or the highest value out of the solutions. However, this can lead to bias in estimation and should be avoided. A better method is to let the data to guide the selection of *τ* by choosing the value of *τ* out of the possible solutions that maximizes the loglikelihood. Therefore the equations are now

$$\tau (\rho ,\eta ,x)=\underset{\tau }{\text{arg}\;\text{max}}\{l\left(\theta |x\right):\rho =\psi_{1}\left(\tau ,\eta \right)\;\text{and}\;\tau h^{\prime }\left(\tau |\eta \right)<-h\left(\tau |\eta \right)\},\;\text{and}$$ (15)

$$\tau (\rho ,\eta ,\alpha ,x)=\underset{\tau }{\text{arg}\;\text{max}}\{l\left(\theta |x\right):\rho =\psi_{2}\left(\tau ,\eta ,\alpha \right)\;\text{and}\;h^{\prime }\left(\tau |\eta \right)<0\}$$ (16)

for composite Pareto I and composite Pareto II models, respectively, where *l*(***θ***|***x***) is the loglikelihood.

This selection method assumes that the ‘true’ solution of *τ* should be the one that gives the highest likelihood. The solution is not to be misunderstood as the maximum likelihood estimate as the number of possible solution of *τ* is limited by the conditions (3) or (6). However, in Bayesian estimation, when the sample size grows, the solution to Eqs (15) and (16) should approach to the maximum likelihood estimates.

### 3.3 Computation to find the value of threshold parameter

Typically, numerical methods have to be used to compute the solution to Eqs (12) and (13). It is also important to consider the condition for the solution of Eqs (12) and (13) when computing the value of *τ*. Namely, the solution must satisfy *τh*′(*τ*|***η***) < −*h*(*τ*|***η***) and *h*′(*τ*|***η***) < 0 for composite Pareto I and II models, respectively. Without considering the restriction on *τ*, the function *ψ*_1_(*τ*, ***η***) or *ψ*_2_(*τ*, ***η***, *α*) may diverge to ±∞, making the numerical computation difficult.

To compute the value of *τ* given the values of other parameters, we use the following functions.

$$\begin{array}{c} \kappa_{1}\left(\tau |\rho ,\eta \right)=\{\begin{array}{cc} -1 & \text{if}\;h(\tau |\eta )=0\;\text{and}\;H(\tau |\eta )=0\\ 1 & \text{if}\;h(\tau |\eta )=0\;\text{and}\;H(\tau |\eta )=1\\ \rho -1 & \text{if}\;\tau h^{\prime }\left(\tau |\eta \right)\geq -h\left(\tau |\eta \right)\\ \rho -\psi_{1}\left(\tau ,\eta \right) & \text{otherwise} \end{array} \end{array}$$ (17)

for composite Pareto I model, and

$$\begin{array}{c} \kappa_{2}\left(\tau |\rho ,\eta ,\alpha \right)=\{\begin{array}{cc} -1 & \text{if}\;h(\tau |\eta )=0\;\text{and}\;H(\tau |\eta )=0\\ 1 & \text{if}\;h(\tau |\eta )=0\;\text{and}\;H(\tau |\eta )=1\\ \rho -1 & \text{if}\;h^{\prime }\left(\tau |\eta \right)\geq 0\\ \rho -\psi_{2}\left(\tau ,\eta ,\alpha \right) & \text{otherwise} \end{array} \end{array}$$ (18)

for composite Pareto II model. The roots of these two functions give the values of *τ* that satisfy Eqs (12) and (13), respectively.

The first two lines in Eqs (17) and (18) are added for numerical stability. These lines are useful when *h*(*τ*|***η***) is computationally 0 and *H*(*τ*|***η***) is computationally 0 or 1. The third lines in Eqs (17) and (18) are useful to impose the restrictions on *τ* as mentioned. The values are set to be *ρ* − 1 since when *τh*′(*τ*|***η***) + *h*(*τ*|***η***) → 0 from the left, then *ψ*_1_(*τ*, ***η***) → 1. Similarly, when *h*′(*τ*|***η***) → 0 from the left, then *ψ*_2_(*τ*, ***η***, *α*) → 1. Setting the values to be *ρ* − 1 makes *κ*_1_(*τ*|*ρ*, ***η***) and *κ*_2_(*τ*|*ρ*, ***η***, *α*) to be continuous functions.

There may be multiple roots to *κ*_1_(*τ*|*ρ*, ***η***) and *κ*_2_(*τ*|*ρ*, ***η***, *α*) which indicates the incidence of multiple *τ* solutions. This proves to be difficult since most root-finding algorithms output only a single root. To find the values of *τ*, we use the uniroot.all function from the rootSolve package in R, which divides the domain of *τ* (for a given lower and upper bounds) into a number of subintervals. The function then detects the changes in the signs of *κ*_1_(*τ*|*ρ*, ***η***) and *κ*_2_(*τ*|*ρ*, ***η***, *α*) on the boundaries of the subintervals, and uses bisection method to find the zeroes at which there is a change in the signs of *κ*_1_(*τ*|*ρ*, ***η***) and *κ*_2_(*τ*|*ρ*, ***η***, *α*).

## 4 Prior distributions for composite Pareto models

In Bayesian setting, prior information on the parameters must be specified and included in the analysis. For composite Pareto models, a closed form for the posterior distributions cannot be found due to the complexity of the likelihood. Thus MCMC method specifically Metropolis–Hastings within Gibbs sampling will be used to estimate the posterior distribution. In this section, focus will be given on using noninformative prior distributions for the parameters. If informative prior distributions were to be used, the posterior estimation process remains the same when using the MCMC algorithm.

Additionally, we assume independence between parameters such that the prior distribution can be written as follows:

p(τ,η,α,β)=p(τ)p(η)p(α,β),

when using prior distribution on *τ*, or:

p(ρ,η,α,β)=p(ρ)p(η)p(α,β), (19)

when using prior distribution on *ρ*.

### 4.1 Prior on *τ* versus prior on *ρ*

Setting a prior information on *τ* can be tricky unless there is a good prior information that can be used. Informative prior in the form of normal distribution has been proposed by Behrens et al. [16] but selecting the hyperparameters can be difficult. When no information on *τ* is available, researchers may opt to use noninformative priors. In this case, a uniform prior can be used for *τ* as proposed by Behrens et al. [16] and Cabras and Castellanos [18]. But as will be shown in the Section 5, using uniform prior on *τ* is not recommended. Villa [19] on the other hand has proposed to use data-dependent prior on *τ* by using Kullback–Leibler divergence to determine the order statistics’ ‘worth’.

In many cases, specifying prior information on *ρ* is easier as compared to specifying prior information on *τ*. This is particularly clear because *ρ* is bounded between 0 and 1 (whereas *τ* is not bounded above) and that *ρ* is free of unit. Furthermore, in many applications that uses generalized Pareto distribution to estimate extreme quantiles, researchers typically choose the threshold value based on the quantiles (e.g. 90th, 99th, 99.5th percentiles). Therefore using prior on *ρ* is more natural than specifying information on some arbitrary threshold value. Informative prior in the form of a beta distribution can be used for *ρ* when required.

**Fig 1:**
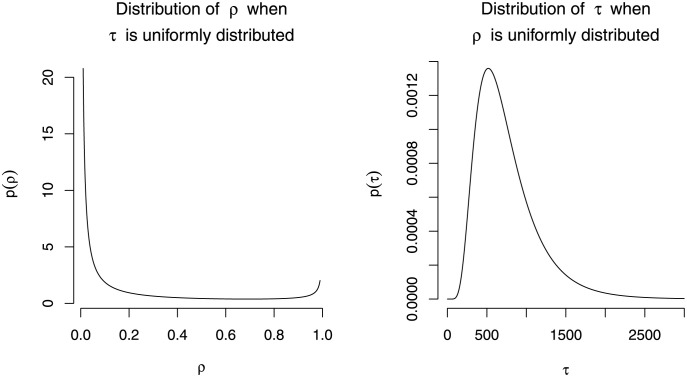
The distribution of *ρ* when *τ* is uniformly distributed (left), and the distribution of *τ* when *ρ* is uniformly distributed (right), when *ρ* = 1 − *H*(*τ*|*η*) with *h*(*x*|*η*) is lognormal distribution with log-mean 6.5, log-variance 0.5^2^.

If there are no information on both *τ* and *ρ*, we can use uniform prior for either *τ* or *ρ*. Note that the distribution of *τ* affects the distribution of *ρ* and vice versa. Fig 1 illustrates an example on how the distribution of *τ* affects the distribution of *ρ* and vice versa. The figure is based on having *ρ* = 1 − *H*(*τ*|***η***) as in the case of bulk models with *h*(*x*|***η***) be a lognormal distribution with log-mean 6.5 and log-variance 0.5^2^. In this case, when *τ* is uniformly distributed, *p*(*ρ*) ∝ 1/*h*(*H*^−1^(1 − *ρ*)|***η***), and when *ρ* is uniformly distributed, *p*(*τ*) = *h*(*τ*|***η***). It can be seen from the figure that when *τ* is uniformly distributed, the distribution of *ρ* is concentrated around 0. On the other hand, when the *ρ* is uniformly distributed between 0 and 1, the distribution of *τ* is unimodal and its density decreases as *τ* increases.

Note that if *X* is a random variable with pdf *f*_*X*_(*x*), *Y* = *v*(*X*) is a function of *X*, and *v*(*X*) is monotonic, then

$$\begin{array}{c} f_{Y}\left(y\right)=f_{X}\left(v^{-1}\left(y\right)\right)|\frac{\partial }{\partial y}v^{-1}\left(y\right)|, \end{array}$$

where *f*_*Y*_(*y*) is the pdf of *Y*. In the case of *ρ* and *τ*, the above equation translates to

$$\begin{array}{c} p_{\tau }\left(\tau \right)=p_{\rho }\left(v^{-1}\left(\tau \right)\right)|\frac{\partial }{\partial \tau }v^{-1}\left(\tau \right)|, \end{array}$$ (20)

where *τ* = *v*(*ρ*) for some function *v*(⋅), if it exists. Assuming the independence between ***η***, (*α*, *β*), and *τ*, the prior can then be written as

$$\begin{array}{cc} p(\tau ,\eta ,\alpha ,\beta ) & =p(\eta )p(\alpha ,\beta )p(\tau )\\ & =p\left(\eta \right)p\left(\alpha ,\beta \right)p\left(v^{-1}\left(\tau \right)\right)|\frac{\partial }{\partial \tau }v^{-1}\left(\tau \right)|\\ & =p\left(\eta \right)p\left(\alpha ,\beta \right)p\left(\rho \right)|\frac{\partial \rho }{\partial \tau }| \end{array}$$ (21)

Using Eq (21), the MCMC algorithm can be performed by sampling *τ* with the prior on *ρ* specified using *p*(*ρ*), which gives equivalent result to the MCMC that samples *ρ* using the prior written in Eq (19). However, it is important to note that this is only valid when the relationship between *ρ* and *τ* is one-to-one, and there is still a need to check for the uniqueness of *τ* given the values of *ρ* and other parameters, for example by using Theorem 1 or 2. In the case of multiple *τ* solutions, the mapping *ρ* ↦ *τ* is not even a function since the relation is one-to-many. In this case, the function *v*(⋅) does not exist and Eq (20) cannot be used.

**Fig 2:**
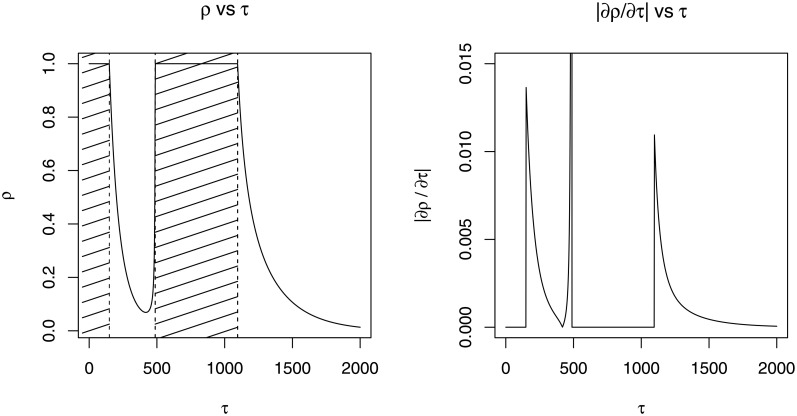
The relationship between *ρ* and *τ* when using a two-component lognormal mixture model for the lower data distribution.

Not only that Eq (20) is invalid for the cases with multiple *τ* solution, specifying the prior according to the equation may lead to poor result on the posterior estimation. As an example, consider using a two-component lognormal mixture model for the lower data distribution, i.e. $h\left(x|\eta \right)=r_{1}f_{LN}\left(x|\mu_{1},\sigma_{1}^{2}\right)+r_{2}f_{LN}\left(x|\mu_{2},\sigma_{2}^{2}\right),$ in a composite Pareto I model with ***r*** = (0.5, 0.5), ***μ*** = (5, 7), and ***σ*** = (0.6, 0.3). The relationship between *ρ* and *τ* is shown in Fig 2. The shaded area in the figure signifies the area where *τh*′(*τ*|***η*** ≥ −*h*(*τ*|***η***) in which the acceptance probability for the Metropolis–Hastings step is zero. Suppose the true value of *τ* is greater than 1000. Then the MCMC that samples *τ* using prior specification on *ρ* and Eq (21) may get stuck in the region between 150 and 500 due to the area with zero acceptance probability, which in turn will cause the parameter point estimates to have huge error. Therefore, it is advised to avoid setting the prior as in Eq (21) for the cases where the relationship between *τ* and *ρ* is not proven to be one-to-one.

Following Cabras and Castellanos [18], it is assumed that *x*_(*m*+1)_ ≤ *τ* ≤ *x*_(*n*−2)_ where *x*_(*i*)_ is the *i*th order statistic, *m* is the dimension of ***η***, and *n* is the sample size. This assumption is required to obtain proper posterior distribution over the parameters ***η***, *α*, and *β*.

### 4.2 Prior distributions for *α* and *β*

For smooth composite Pareto I model, and continuous bulk model with Pareto I tail, the value of *α* is determined by Eqs (2) or (10). Therefore in these cases, prior distribution on *α* needs not to be specified since *α* is not a free parameter. However, when the composite Pareto I model is not assumed to be differentiable, or when the pdf of bulk model with Pareto I tail is not assumed to be continuous, the following prior distribution can be used:

$$\begin{array}{c} p\left(\alpha \right)\propto \frac{1}{\alpha }, \end{array}$$

which is the Jeffreys prior for *α* for a Pareto I distribution with known threshold or scale parameter.

For composite Pareto II model with smooth pdf and bulk model with Pareto II tail and continuous pdf, the value of *β* is specified by Eqs (5) or (11). Since in this case *α* is a free parameter, a prior distribution on *α* is required. The Jeffreys prior for *α* for a Pareto II distribution when the parameter *β* and the threshold or location parameter are fixed or known as written below is used:

$$\begin{array}{c} p\left(\alpha \right)\propto (\frac{1}{\alpha +1})(\frac{1}{\alpha +2}). \end{array}$$

However, when the composite Pareto II model is not assumed to be differentiable, or the discontinuous bulk model with Pareto II tail is used, both *α* and *β* are free parameters and the prior distributions on *α* and *β* are required. Cabras and Castellanos [18] have proposed to use the Jeffreys prior for a Pareto II distribution with fixed or known location threshold as written below:

$$\begin{array}{c} p\left(\alpha ,\beta \right)\propto \frac{1}{\beta }(\frac{1}{\alpha +1})(\frac{1}{\alpha (\alpha +2)}{)}^{1/2}. \end{array}$$ (22)

Using this prior leads to proper posterior distribution [23]. This prior distribution is used in our analysis.

### 4.3 Prior distributions for *η*

The prior distribution for ***η*** depends on the choice of lower data distribution *h*(*x*|***η***). Default priors can be use when no information is available. For example, if *h*(*x*|***η***) is a lognormal distribution with log-mean *μ* and log-variance *σ*^2^, then ***η*** = (*μ*, *σ*^2^). As recommended by Cabras and Castellanos [18], the following default prior is used:

$$\begin{array}{c} p\left(\mu ,\sigma^{2}\right)\propto \frac{1}{\sigma^{2}}. \end{array}$$

## 5 Simulation study

In this section, the performance of posterior point estimates when using uniform prior on *τ* and using uniform prior on *ρ* is compared. To study the performance of these two prior choices, eight simulation scenarios are used. Four of these scenarios involve composite Pareto I models, and the other four involve composite Pareto II models. For the lower data distribution, we use lognormal distribution with log-mean 6.5 and log-variance 0.5^2^. Table 1 presents the eight simulation scenarios used as well as the values for the parameters. The choice of parameter values and lower data distribution is arbitrary as similar results are obtained when using other values or distributions.

**Table 1:** Simulation scenarios and their true parameters.

Scenario	Pareto type	Model	Assumptions		True param.					
			Cont.	Diff.	*μ*	*σ*	*τ*	*ρ*	*α*	*β*
LNP1	I	General	Yes	Yes	6.5	0.5	1300	0.1176	2.681	-
LNP1.nodiff	I	General	Yes	No	6.5	0.5	1300	0.1176	2.681	-
LNP1B	I	Bulk	Yes	No	6.5	0.5	1300	0.0901	3.608	-
LNP1B.nocont	I	Bulk	No	No	6.5	0.5	1300	0.0901	3.608	-
LNP2	II	General	Yes	Yes	6.5	0.5	1300	0.1196	2.5	1236.3
LNP2.nodiff	II	General	Yes	No	6.5	0.5	1300	0.1196	2.5	1236.3
LNP2B	II	Bulk	Yes	No	6.5	0.5	1300	0.0901	2.5	900.8
LNP2B.nocont	II	Bulk	No	No	6.5	0.5	1300	0.0901	2.5	900.8

General models follow Section 2.1 and 2.2. Bulk models follow Section 2.3.

Referring to Table 1, in the first two scenarios, i.e. LNP1 and LNP1.nodiff, the parameters used are exactly the same. Here, LNP1 assumes the pdf to be differentiable while LNP1.nodiff does not assume the pdf to be differentiable. Therefore in LNP1, *α* is a function of other parameters as in Eq (2) and prior distribution is not required for estimation. Meanwhile, LNP1.nodiff requires prior distribution on *α* for estimation as *α* is a free parameter. Similarly, LNP2 assumes differentiable pdf while LNP2.nodiff does not, LNP1B assumes continuous pdf while LNP1B.nocont does not, and lastly LNP2B also assumes continuous pdf while LNP2B.nocont does not assume continuous pdf.

To compare the performance between the two priors, 100 datasets with *n* = 1000 observations each are generated for each scenarios using the parameters as shown in Table 1. For each dataset, Metropolis-within-Gibbs algorithm with 50 000 iterations, where the first 20 000 iterations are treated as burn-in period, is used to estimate the posterior distribution. The convergence of the MCMC are checked using Gelman–Rubin diagnostic. Then, for each dataset, the posterior mean, median, and mode of *τ* are estimated and the root mean square error (RMSE) is calculated. The RMSE of the point estimates based on the posterior densities are compared in order to investigate which prior gives a more accurate estimate.

Let *τ*_*a*_ be the posterior samples of *τ* obtained when using uniform prior on *τ* and *τ*_*b*_ be the posterior samples of *τ* obtained when using uniform prior on *ρ*. Table 2 shows the RMSE of the posterior mean, median and mode of *τ*_*a*_ and *τ*_*b*_ for the eight simulation scenarios. The last column on the table also shows the mean of the posterior standard deviation. From Table 2, it can be seen that the RMSE for mean, median, and mode is lower for *τ*_*b*_ compared to *τ*_*a*_, for all scenarios. This implies that the point estimates using uniform prior on *ρ* has lower bias compared to point estimates obtained when using uniform prior on *τ*. Additionally, it can be observed that the mean of standard deviation of *τ*_*a*_ is higher than the mean of standard deviation of *τ*_*b*_ for all scenarios. Therefore the posterior distribution *τ*_*a*_ generally is more spread compared to the posterior distribution *τ*_*b*_.

**Table 2:** Root mean square error (RMSE) of the posterior mean, median and mode when using uniform prior on *τ* versus uniform prior on *ρ*.

Scenario		RMSE (Mean)	RMSE (Median)	RMSE (Mode)	Mean of s.d.
LNP1	*τ*_*a*_	195.5	166.0	134.8	161.8
	*τ*_*b*_	133.6	126.3	119.0	127.4
LNP1.nodiff	*τ*_*a*_	518.7	530.6	601.6	452.0
	*τ*_*b*_	227.9	238.0	308.0	339.1
LNP1B	*τ*_*a*_	486.9	430.5	431.3	401.5
	*τ*_*b*_	198.1	170.0	147.2	219.4
LNP1B.nocont	*τ*_*a*_	717.8	749.6	936.5	503.7
	*τ*_*b*_	216.0	230.5	317.7	342.2
LNP2	*τ*_*a*_	343.4	320.6	290.2	289.7
	*τ*_*b*_	214.1	216.3	231.5	227.7
LNP2.nodiff	*τ*_*a*_	684.5	706.8	773.6	545.2
	*τ*_*b*_	344.1	386.9	479.1	421.7
LNP2B	*τ*_*a*_	412.9	402.4	438.2	370.7
	*τ*_*b*_	203.8	214.4	261.2	235.7
LNP2B.nocont	*τ*_*a*_	626.9	651.4	775.0	444.3
	*τ*_*b*_	206.2	230.0	379.7	328.9

*τ*_*a*_ is the posterior samples of *τ* when using uniform prior on *τ*. *τ*_*b*_ is the posterior samples of *τ* when using uniform prior on *ρ*.

The simulations are repeated but with *τ* = 1850, a more extreme value compared to previous simulations with *τ* = 1300. The values of the parameters are given in Table 3. In these cases, only around 2% of the data come from Pareto tail which translate to around 20 observations out of *n* = 1000. Using the given parameter values, 100 datasets are generated and the RMSE of the posterior mean, median and mode are calculated and given in Table 4. Similar results are observed in the table: first, the point estimates found from using uniform prior on *ρ* give lower bias compared to point estimates found from using uniform prior on *τ* and second, the posterior distribution of *τ* obtained from using uniform prior on *τ* is more spread with higher standard deviation compared to the posterior distribution of *τ* obtained from using uniform prior on *ρ*. Comparing the results obtained when *τ* = 1300 in Table 2, it appears that the relative differences of the RMSE found for the point estimates of *τ*_*a*_ and *τ*_*b*_ are much higher when *τ* = 1850, as seen in Table 4.

**Table 3:** Simulation scenarios and their true parameters for the second round of simulations with *τ* = 1850.

Scenario	Pareto type	Model	Assumptions		True param.					
			Cont.	Diff.	*μ*	*σ*	*τ*	*ρ*	*α*	*β*
LNP1x	I	General	Yes	Yes	6.5	0.5	1850	0.0240	4.092	-
LNP1.nodiffx	I	General	Yes	No	6.5	0.5	1850	0.0240	4.092	-
LNP1Bx	I	Bulk	Yes	No	6.5	0.5	1850	0.0204	4.828	-
LNP1Bx.nocont	I	Bulk	No	No	6.5	0.5	1850	0.0204	4.828	-
LNP2x	II	General	Yes	Yes	6.5	0.5	1850	0.0269	2.5	1271.7
LNP2x.nodiff	II	General	Yes	No	6.5	0.5	1850	0.0269	2.5	1271.7
LNP2Bx	II	Bulk	Yes	No	6.5	0.5	1850	0.0204	2.5	958.0
LNP2Bx.nocont	II	Bulk	No	No	6.5	0.5	1850	0.0204	2.5	958.0

General models follow Section 2.1 and 2.2. Bulk models follow Section 2.3.

**Table 4:** Root mean square error (RMSE) of the posterior mean, median and mode when using uniform prior on *τ* versus uniform prior on *ρ* for the second round of simulations.

Scenario		RMSE (Mean)	RMSE (Median)	RMSE (Mode)	Mean of s.d.
LNP1x	*τ*_*a*_	881.9	846.6	830.3	398.5
	*τ*_*b*_	562.1	521.0	442.9	287.6
LNP1x.nodiff	*τ*_*a*_	1009.9	1065.5	1305.5	562.4
	*τ*_*b*_	408.9	326.3	237.3	462.5
LNP1Bx	*τ*_*a*_	744.8	730.8	804.7	412.6
	*τ*_*b*_	400.5	361.5	286.6	307.9
LNP1Bx.nocont	*τ*_*a*_	898.6	952.6	1110.7	427.3
	*τ*_*b*_	423.4	420.0	430.9	389.2
LNP2x	*τ*_*a*_	885.4	866.8	911.6	531.5
	*τ*_*b*_	400.6	382.9	372.2	399.0
LNP2x.nodiff	*τ*_*a*_	1222.5	1300.4	1534.1	647.4
	*τ*_*b*_	513.3	481.5	521.6	581.8
LNP2Bx	*τ*_*a*_	712.6	708.9	818.4	505.8
	*τ*_*b*_	271.7	290.1	370.2	375.4
LNP2Bx.nocont	*τ*_*a*_	999.9	1068.1	1205.6	467.6
	*τ*_*b*_	387.3	417.8	551.9	468.8

*τ*_*a*_ is the posterior samples of *τ* when using uniform prior on *τ*. *τ*_*b*_ is the posterior samples of *τ* when using uniform prior on *ρ*.

**Fig 3:**
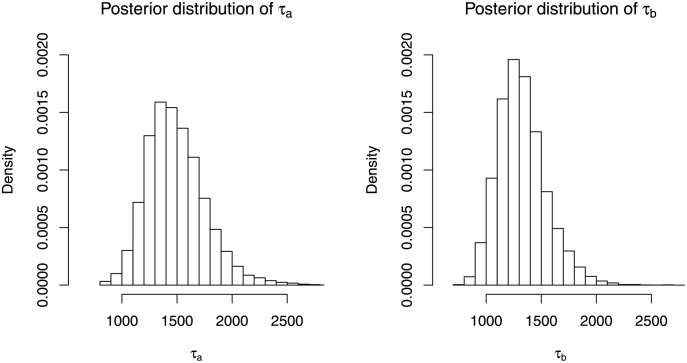
Posterior distribution estimates of *τ*_*a*_ and *τ*_*b*_ under LNP2 scenario using the same data.

One possible explanation on the poor performance when using uniform prior on *τ* is that the prior assumes equal probability of *τ*, including for very high values of *τ*. We believe this shifts the posterior estimate of *τ* to the extreme values, sometimes close to the highest possible value, *x*_(*n*−2)_, as we assume *x*_(*m*+1)_ ≤ *τ* ≤ *x*_(*n*−2)_. This result if further illustrated using Fig 3 which shows the posterior distributions of *τ*_*a*_ and *τ*_*b*_ under LNP2 scenario using the same data. It can be seen in the figure that the posterior distribution of *τ*_*a*_ has longer tail to the right compared to the posterior distribution of *τ*_*b*_. This behaviour is commonly observed in all scenarios considered, causing the standard deviation of *τ*_*a*_ to be higher than the standard deviation of *τ*_*b*_, as shown in the last column in Tables 2 and 4.

In addition, the posterior obtained from using uniform prior on *τ* may get worse when *x*_(*n*−2)_ increases, typically when sample size increases or when the underlying pdf exhibits very heavy tail properties. In our simulation, increasing the value of *τ* increases the heaviness of the upper tail and as a result, the performance of Bayesian approach using uniform prior on *τ* becomes worse as seen in Tables 2 and 4. It is thus not recommended to use uniform prior on *τ* for very heavy tail data since posterior distribution of *τ* may be overestimated. Thus. using uniform prior on *ρ* is better when little or no information is available regarding the parameter *ρ* and *τ*.

## 6 Applications

### 6.1 Application to income distribution

The composite Pareto I model is applied to the Malaysian Household Income Survey (HIS) to study the income distribution in Malaysia. Twice every five years, the HIS is conducted by Department of Statistics Malaysia (DOSM) to gather information regarding the economic well-being of Malaysian households. Surveys are performed by interviewing randomly selected households and several information including the household income and expenditure are recorded. Five HIS datasets are used: year 2007, 2009, 2012, 2014 and 2016. These data are provided by DOSM and Bank Data UKM, which consist of subsets of the whole survey data. (Unfortunately, access to the data is restricted and cannot be shared publicly. However, these data can be requested online from https://www.dosm.gov.my/v1/index.php.). There are more than 12 000 observations in each dataset, representing the households in Malaysia.

**Fig 4:**
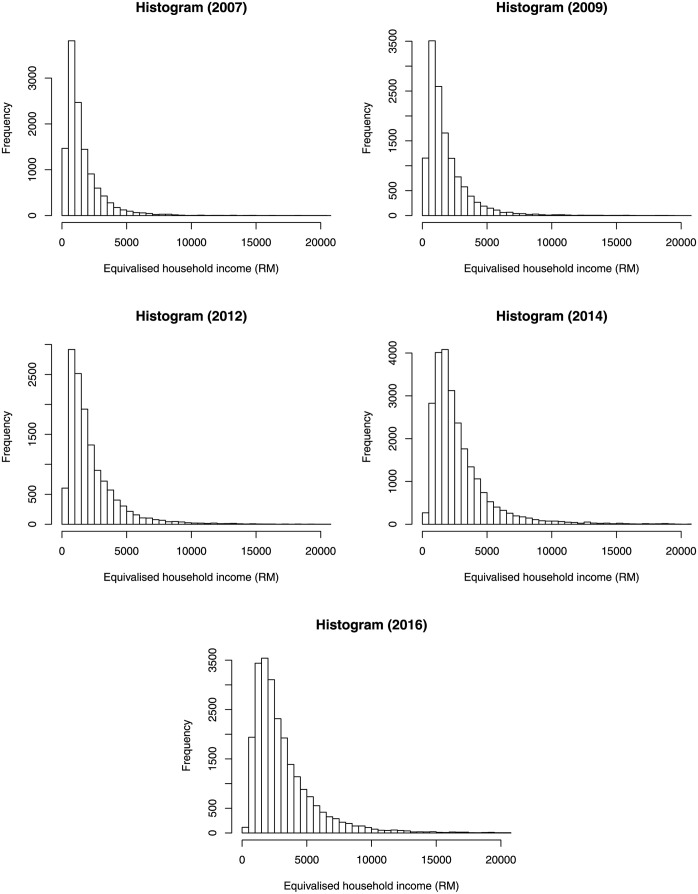
Histograms of the equivalised household income in Ringgit Malaysia (RM) for the year 2007, 2009, 2012, 2014 and 2016.

Since two households with the same income may not be in the same economic condition due to the difference in the size of household, the monthly gross income is equivalised by dividing it by the square root of the size of household. This square root equivalence scale is commonly used in studies including Congressional Budget Office [24] and OECD [25]. Although households are given weight depending on their location and strata, for simplicity, the weights are not used in our application. Fig 4 shows the histograms of the five datasets.

These data sets have been studied previously by various authors [26–30]. For example, Razak and Shahabuddin [26] have noted that the upper tail of the Malaysian household income fits the Pareto tail behaviour. Safari et al. [27] on the other hand have applied a semi-parametric approach to model the income distribution by combining Pareto type I distribution for the upper tail and a nonparametric approach for the rest of the data. In a recent work, the lognormal-Pareto II composite model was found to be able to describe the income distribution quite well [31].

We apply the smooth composite Pareto I model to these datasets and the two-component lognormal mixture distribution is used for the lower data distribution. In this case,

$$\begin{array}{c} h\left(x|\eta \right)=rf_{LN}\left(x|\mu_{1},\sigma_{1}^{2}\right)+\left(1-r\right)f_{LN}\left(x|\mu_{2},\sigma_{2}^{2}\right), \end{array}$$

where ***η*** = (*r*, *μ*_1_, *μ*_2_, *σ*_1_, *σ*_2_) and *r* ∈ (0, 1) is the weight of the first component and *f*_*LN*_(*x*|*μ*, *σ*^2^) is the pdf of lognormal distribution with log-mean *μ* and log-variance *σ*^2^. This choice of lower data distribution does not guarantee unique solution of *τ* given *ρ* and other parameters. However, when implemented to the datasets using the MCMC algorithm, no instances of multiple *τ* solutions is found.

For the prior distributions, reference prior is used for the parameters of the lognormal distribution such that $p\left(\mu_{j},\sigma_{j}^{2}\right)\propto 1/\sigma_{j}^{2}$ and uniform prior is used for *r*. Using this real data, the Bayesian analysis is performed twice by using uniform prior on *τ* and uniform prior on *ρ* to compare the two results.

The estimated posterior means of *τ* and *ρ* are given in Table 5 along with the *p*-values of Kolmogorov–Smirnov (KS) goodness-of-fit test using the estimated parameter values. From the table, around 2% to 7% of the observations come from the Pareto tail. It can also be seen that the estimated *τ* obtained by using uniform prior on *ρ* are smaller compared to estimated *τ* obtained by using uniform prior on *τ*, which is similar to the results obtained in the simulation study. This may indicate that using uniform prior on *τ* overestimates the threshold value.

**Table 5:** Estimates of *τ* and *ρ* for Malaysian household income distribution. Included in the table is the *p*-value obtained from KS test using the estimated parameters.

Year	Uniform prior on *τ*			Uniform prior on *ρ*		
	$\hat{\tau }$	$\hat{\rho }$	*p*-value	$\hat{\tau }$	$\hat{\rho }$	*p*-value
2007	6150	0.0219	0.9157	5979	0.0236	0.9278
2009	7172	0.0201	0.9539	6881	0.0221	0.9756
2012	7385	0.0312	0.5971	7214	0.0332	0.6376
2014	6261	0.0717	0.2186	6157	0.0745	0.1057
2016	9536	0.0323	0.0298	9433	0.0331	0.3245

Additionally, the *p*-values from the KS test are higher when using uniform prior on *ρ* rather than uniform prior on *τ*, except for the year 2014 in which the *p*-value is higher when using uniform prior on *τ*. The *p*-value indicates that except for the year 2014, the estimated parameters obtained when using uniform prior on *ρ* fit the datasets better compared to the estimated parameters obtained when using uniform prior on *τ*. Therefore in general, using uniform prior on *ρ* is deemed better when compared to using uniform prior on *τ* in this application.

### 6.2 Application to NASDAQ-100

**Fig 5:**
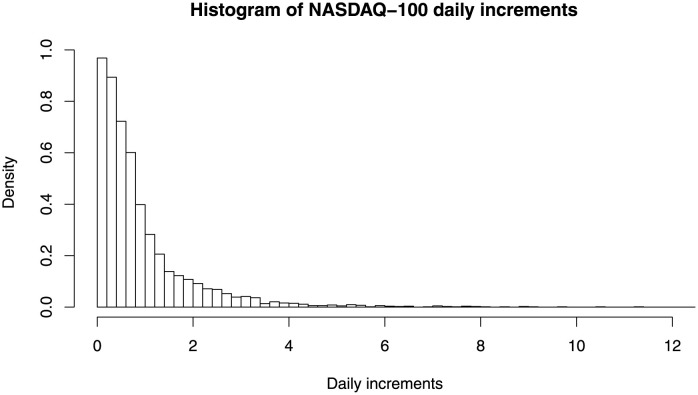
Histogram of the NASDAQ-100 daily increments used in application.

We implement the composite Pareto model to NASDAQ-100, a stock market index made up of 100 largest companies listed in the Nasdaq stock exchange. The daily increments of the index are taken from Yahoo Finance for the period of January 1985 to May 2002 with the number of days *n* = 4394. The daily increment values are transformed such that

$$\begin{array}{c} y_{t}=|\frac{P_{t}}{P_{t-1}}-1|\times 100,\;t=1,\ldots ,4394, \end{array}$$

where *P*_*t*_ is the adjusted closing price for the index at day *t*. Fig 5 shows the histogram of the NASDAQ-100 daily increments. From the figure, it is clear that the distribution exhibits heavy tail behaviour, thus using composite Pareto model can be beneficial.

This data has been analyzed previously by Behrens et al. [16] and Villa [19] using bulk model with Pareto type II tail and gamma distribution. For the threshold parameter, Behrens et al. [16] have used uniform prior distribution whereas Villa [19] has used prior distribution based on the Kullback–Leibler divergence. In our implementation, we use both uniform prior on *τ* and uniform prior on *ρ* and compare our results with the two literatures.

We apply the same bulk model without the continuity condition to the data. In our application, the lower data distribution is *h*(*x*|***η***) = *f*_*G*_(*x*|*a*, *b*) where *f*_*G*_(*x*|*a*, *b*) is the pdf of a gamma distribution with shape parameter *a* and rate parameter *b*. Reference prior is used for *a* and *b* such that

$$\begin{array}{c} p\left(a,b\right)\propto \frac{1}{b}\sqrt{\frac{a\psi^{\left(1\right)}\left(a\right)-1}{a}}, \end{array}$$

where *ψ*^(1)^(⋅) is the trigamma function. The prior distribution for *α* and *β* is given in Eq (22). Additionally, the uniform prior on *τ* and uniform prior on *ρ* are used for comparing the results based on these these two choices of prior, along with the results obtained by Behrens et al. [16] and Villa [19]. Note that both of these literatures have used informative prior for the shape and rate parameters but the values for the hyperparameters are not provided. Therefore, we are unable to duplicate their prior for our application and we opt to use reference prior instead.

Behrens et al. [16] and Villa [19] have used a different parameterization to the model. Let *ξ* = 1/*α* and *σ* = *β*/*α*. Table 6 shows the estimated posterior mode for the Pareto II tail along with the estimated parameters from Behrens et al. [16] and Villa [19]. Note that the estimate for *ρ* is not given in both Behrens et al. [16] and Villa [19] thus left empty in Table 6. From the table, around 30% of the observations come from the Pareto II distribution and the other 70% come from gamma distribution. The parameter estimates using uniform prior on *τ* is similar to the parameter estimates using uniform prior on *ρ*, indicating that there are not much advantages of using uniform prior on *ρ* as opposed to uniform prior on *τ* as seen in the previous simulations and example.

**Table 6:** Parameter estimates for NASDAQ-100 application with the 95% credible interval in bracket.

	Uniform prior on *τ*	Uniform prior on *ρ*	Behrens et al.	Villa
$\hat{\tau }$	0.9356 (0.86, 0.96)	0.9354 (0.86, 0.96)	0.9619 (0.79, 1.13)	0.93 (0.91, 0.94)
$\hat{\xi }$	0.1497 (0.09, 0.22)	0.1498 (0.09, 0.22)	0.1567 (0.09, 0.23)	0.15 (0.08, 0.21)
$\hat{\sigma }$	0.9722 (0.88, 1.06)	0.9565 (0.88, 1.06)	0.9735 (0.86, 1.08)	0.98 (0.90, 1.06)
$\hat{\rho }$	0.3032 (0.29 0.34)	0.3042 (0.29, 0.34)		

Both parameter estimates found using uniform prior on *τ* and uniform prior on *ρ* are consistent to those found by Behrens et al. [16] and Villa [19]. Although Behrens et al. [16] also used uniform prior for the threshold parameter, the differences between our results and theirs might be due to the difference prior distribution used for the parameter of the gamma distribution. As mentioned previously, the values of hyperparameters used are not provided by Behrens et al. [16] and thus we cannot duplicate their results. Additionally, the credible intervals found in our analysis is narrower than the credible interval found by Behrens et al. [16], but broader when compared to the results in Villa [19]. It is noted that Villa [19] has used data dependent prior distribution for the threshold parameter.

## 7 Conclusion

When using Bayesian approach to composite Pareto models, the prior distribution for the threshold parameter *τ* must first be specified. However, choosing the prior distribution for *τ* can be difficult. Previous studies opt to use normal or uniform distributions for the prior of *τ*. In our study, we propose to specify the prior using the proportion of data coming from the Pareto distribution which is the parameter *ρ*. The two parameters *ρ* and *τ* are dependent as we can write *ρ* as a function of *τ* and other parameters. Therefore, specifying the prior distribution of *ρ* affects the prior distribution of *τ*.

Given the values of *ρ* and other parameters, the value of *τ* can be obtained numerically. However, the solution of *τ* given *ρ* and other parameters may not be unique for some lower data distributions. We have proven that for commonly used lower data distributions, for example lognormal, gamma, or Weibull distributions, the solution is unique. For distributions that are not covered in the proven theorems, we propose to select the solution *τ* that solves the required equations and maximizes the loglikelihood, out of the possible solutions.

One main benefit of specifying prior on *ρ* instead of *τ* is that it can be more natural and easily interpreted. Historically, researchers have used quantiles in extreme value theory to set up the threshold value that separates lower data distribution and Pareto distribution. Specifying prior on *ρ* is seen as an extension of this approach. Another benefit of using prior on *ρ* rather than *τ* is that *ρ* is bounded between 0 and 1 while *τ* is not bounded above. Hence, it is easier for researchers to specify the prior distribution on *ρ*, for example by using beta distribution, than specifying the prior distribution of *τ*.

When there are no information regarding *τ* or *ρ*, uniform prior can be used for either *τ* or *ρ*. In our simulations, using uniform prior on *ρ* leads to less biased point estimates compared to using uniform prior on *τ*. Using uniform prior on *τ* assumes equal density of *τ* even at extremely high values and may cause overestimation of the parameter. It is thus not recommended to use uniform prior on *τ* but to use uniform prior on *ρ* instead when there is a lack of information regarding *τ* and *ρ*.

## Supporting information

S1 Appendix Proofs for Theorems 1 and 2. (PDF)
