# Supplementary material for: On Bayesian approach to composite Pareto models
Source: PLoS One. 2021 Sep 23;16(9):e0257762. doi: 10.1371/journal.pone.0257762 (PMC8460011; doi:10.1371/journal.pone.0257762)
Supplement: S1 Appendix — (PDF) [file pone.0257762.s001.pdf]

S1 Appendix

Proof of Theorem 1

Proof. Suppose $h'(x|\boldsymbol{\eta}) = h(x|\boldsymbol{\eta})g(x|\boldsymbol{\eta})$ for some function $g(x|\boldsymbol{\eta})$. Then we can write

$$h(\tau|\boldsymbol{\eta}) + \tau h'(\tau|\boldsymbol{\eta}) = h(\tau|\boldsymbol{\eta})(1 + \tau g(\tau|\boldsymbol{\eta}))$$

and

$$\rho = \frac{\tau h(\tau|\boldsymbol{\eta})}{\tau h(\tau|\boldsymbol{\eta}) - H(\tau|\boldsymbol{\eta})(1 + \tau g(\tau|\boldsymbol{\eta}))}.$$

Taking the partial derivative with respect to τ gives,

$$\frac{\partial \rho}{\partial \tau} = \frac{C}{[\tau h(\tau|\boldsymbol{\eta}) - H(\tau|\boldsymbol{\eta})(1 + \tau g(\tau|\boldsymbol{\eta}))]^2},$$

where

$$\begin{aligned} C &= [\tau h(\tau|\boldsymbol{\eta}) - H(\tau|\boldsymbol{\eta})(1 + \tau g(\tau|\boldsymbol{\eta}))] \frac{\partial}{\partial \tau}\{\tau h(\tau|\boldsymbol{\eta})\} - \tau h(\tau|\boldsymbol{\eta}) \frac{\partial}{\partial \tau}\{\tau h(\tau|\boldsymbol{\eta}) - H(\tau|\boldsymbol{\eta})(1 + \tau g(\tau|\boldsymbol{\eta}))\} \\ &= \tau h(\tau|\boldsymbol{\eta})^2[1 + \tau g(\tau|\boldsymbol{\eta})] - h(\tau|\boldsymbol{\eta})H(\tau|\boldsymbol{\eta})[1 + \tau g(\tau|\boldsymbol{\eta})]^2 + \tau h(\tau|\boldsymbol{\eta})H(\tau|\boldsymbol{\eta})[g(\tau|\boldsymbol{\eta}) + \tau g'(\tau|\boldsymbol{\eta})] \end{aligned}$$

after some simplifications. Note that $h(\tau|\boldsymbol{\eta}) + \tau h'(\tau|\boldsymbol{\eta}) = h(\tau|\boldsymbol{\eta})[1 + \tau g(\tau|\boldsymbol{\eta})] < 0$ is a condition for $\alpha > 0$, where α is given by Eq (2). Then if $g(\tau|\boldsymbol{\eta}) + \tau g'(\tau|\boldsymbol{\eta}) < 0$, then it follows that C is a sum of negative values resulting $C < 0$ and

$$\frac{\partial \rho}{\partial \tau} < 0.$$

Therefore the solution to

$$\tau(\boldsymbol{\eta}, \rho) = \left\{\tau : \rho = \frac{\tau h(\tau|\boldsymbol{\eta})^2}{\tau h(\tau|\boldsymbol{\eta})^2 - H(\tau|\boldsymbol{\eta})[h(\tau|\boldsymbol{\eta}) + \tau h'(\tau|\boldsymbol{\eta})]} \text{ and } \tau h'(\tau|\boldsymbol{\eta}) < -h(\tau|\boldsymbol{\eta})\right\}$$

must be unique. □

Proof of Theorem 2

Proof. Suppose $h'(x|\boldsymbol{\eta}) = h(x|\boldsymbol{\eta})g(x|\boldsymbol{\eta})$ for some function $g(x|\boldsymbol{\eta})$. Then,

$$\begin{aligned} \rho &= \frac{(\alpha+1)h(\tau|\boldsymbol{\eta})^2}{(\alpha+1)h(\tau|\boldsymbol{\eta})^2 - \alpha h'(\tau|\boldsymbol{\eta})H(\tau|\boldsymbol{\eta})} \\ &= \frac{(\alpha+1)h(\tau|\boldsymbol{\eta})}{(\alpha+1)h(\tau|\boldsymbol{\eta}) - \alpha g(\tau|\boldsymbol{\eta})H(\tau|\boldsymbol{\eta})}. \end{aligned}$$

The partial derivative with respect to τ,

$$\frac{\partial \rho}{\partial \tau} = \frac{C}{[(\alpha+1)h(\tau|\boldsymbol{\eta}) - \alpha g(\tau|\boldsymbol{\eta})H(\tau|\boldsymbol{\eta})]^2},$$

where

$$\begin{aligned} C &= [(\alpha+1)h(\tau|\boldsymbol{\eta}) - \alpha g(\tau|\boldsymbol{\eta})H(\tau|\boldsymbol{\eta})]\,[(\alpha+1)h'(\tau|\boldsymbol{\eta})] \\ &= \alpha(\alpha+1)h(\tau|\boldsymbol{\eta})\left[h'(\tau|\boldsymbol{\eta}) + H(\tau|\boldsymbol{\eta})\left[g'(\tau|\boldsymbol{\eta}) - g(\tau|\boldsymbol{\eta})^2\right]\right] \end{aligned}$$

after some simplifications. If $h'(\tau|\boldsymbol{\eta}) + H(\tau|\boldsymbol{\eta})\left[g'(\tau|\boldsymbol{\eta}) - g(\tau|\boldsymbol{\eta})^2\right] < 0$, then

$$\frac{\partial \rho}{\partial \tau} < 0.$$

Therefore the solution to

$$\tau(\rho, \boldsymbol{\eta}, \alpha) = \left\{\tau : \rho = \frac{(\alpha+1)h(\tau|\boldsymbol{\eta})^2}{(\alpha+1)h(\tau|\boldsymbol{\eta})^2 - \alpha h'(\tau|\boldsymbol{\eta})H(\tau|\boldsymbol{\eta})} \text{ and } h'(\tau|\boldsymbol{\eta}) < 0\right\}$$

must be unique. □
